# Beyond motor rescue: defining adequate SMN restoration in spinal muscular atrophy

**DOI:** 10.1172/JCI211991

**Published:** 2026-11-02

**Authors:** Crystal Jing Jing Yeo

**Affiliations:** 1 National Neuroscience Institute, Singapore, Singapore.; 2 Institute of Molecular and Cell Biology (IMCB), Agency for Science, Technology and Research (A*STAR), Singapore, Singapore.; 3 Duke-National University of Singapore Medical School, Singapore, Singapore.; 4 Department of Neurology, Boston Children’s Hospital, Harvard Medical School, Boston, Massachusetts, USA.; 5 Institute of Education in Healthcare and Medical Sciences, School of Medicine, Medical Sciences and Nutrition, University of Aberdeen, Aberdeen, Scotland.

## Abstract

Spinal muscular atrophy (SMA) is characterized by motor neuron degeneration due to deficiency in the survival of motor neuron (SMN) protein. Disease-modifying therapies have transformed outcomes in SMA, while also revealing an increasingly complex spectrum of residual disease biology. In this issue of the *JCI*, Tapken, Rahmel-Stein, and colleagues demonstrated cell-specific SMN expression and persistent alterations in peripheral immune cell populations in nusinersen- or risdiplam-treated adults with SMA. Their findings extend the question of selective vulnerability beyond motor neurons and raise an increasingly important therapeutic question: What constitutes adequate SMN restoration? This Commentary considers how cell type, developmental timing, SMN requirement, and downstream response may shape treatment-modified SMA, with implications for systemic and CNS-directed therapies.

Spinal muscular atrophy (SMA) is a neuromuscular disease involving progressive degeneration of the spinal motor neurons, leading to weakening and wasting of skeletal muscle. It is caused by a deficiency in full-length survival motor neuron (SMN) protein in motor neurons. Pathogenic mutations in the *SMN1* gene encoding a truncated SMN protein underlie this deficiency, and disease severity and age of onset vary widely depending on the specific *SMN1* mutation. Therapeutic approaches for SMA involve increasing the availability of SMN protein using a variety of gene therapy approaches, including restoring *SMN1* function (onasemnogene abeparvovec) and modification of *SMN2* alternative splicing to enhance its contribution to full-length SMN levels (nusinersen, risdiplam). Nusinersen, risdiplam, and onasemnogene abeparvovec have profoundly improved survival and motor outcomes in individuals with SMA (1) (Figure 1A).

The therapeutic transformation of SMA is one of the major successes of precision neuromuscular medicine. Yet this success has brought a longstanding biological paradox into sharper focus: How does deficiency of the ubiquitously expressed SMN protein produce such striking cell- and tissue-specific vulnerability?

Motor neurons provided an early paradigm for this concept. Ruggiu et al. showed that motor neurons intrinsically produce fewer full-length *SMN2* transcripts than do other spinal cord cells because of less efficient exon 7 inclusion, potentially placing them closer to a critical threshold of SMN deficiency (2). Rodriguez-Muela et al. subsequently demonstrated substantial heterogeneity in SMN expression, even among motor neurons sharing the same genetic background (3). Motor neurons with lower SMN levels are more likely to degenerate, whereas those maintaining higher SMN expression are relatively resistant, and increasing SMN expression promotes survival (3). Together, these studies established an important principle: Vulnerability reflects the relationship between SMN availability and the requirements of an individual cell.

## SMN deficiency and its effects in immune cells

In this issue of the *JCI*, Tapken, Rahmel-Stein, and co-authors extend this principle beyond the motor neuron to the immune system (4). In neonatal SMA mice, B cells were markedly depleted, DCs were reduced, and neutrophils and macrophages were expanded relative to that seen in control littermates. B cells and macrophages were among the populations with the highest SMN expression in both SMA and control mice. In human peripheral blood from healthy controls, DCs and monocytes expressed particularly high SMN levels. Compared with healthy controls, adults with SMA showed altered B, T, and NK cell populations, together with broadly reduced SMN expression in immune cells, albeit with DCs and monocytes still retaining the highest SMN levels (4). The authors propose that immune cell populations with intrinsically high SMN expression may have greater SMN requirements and become vulnerable when SMN falls below a critical threshold. Their monocyte differentiation experiments further suggest that the cellular differentiation state itself may alter SMN-dependent RNA-processing requirements.

More recently, Leow et al. demonstrated that hepatocyte-intrinsic SMN deficiency causes mitochondrial dysfunction, impaired fatty acid metabolism, and hepatic steatosis, with transcriptional changes extending beyond metabolism to other hepatic functions, including complement regulation (5). Notably, the degree of steatosis was not proportional to the magnitude of SMN deficiency, suggesting that SMN abundance alone does not determine phenotypic severity. Observations across motor neurons, hepatocytes, and immune cells collectively support a more nuanced model in which disease expression reflects the intersection of SMN availability, cell-specific requirement, the developmental or functional state, and the downstream response to SMN loss. The selective vulnerability question in SMA may therefore be evolving from “why motor neurons?” to a broader question: How much SMN does each cell require, when does it require it, and what happens when that requirement is not met? These questions have taken on greater importance because the development of successful therapies has fundamentally altered the natural history of SMA. Patients are surviving longer, treatment increasingly begins in early life due to newborn screening, and phenotypes that were previously obscured by severe weakness or early mortality may become visible (1).

## Extraneuronal outcomes in nusinersen- and risdiplam-treated individuals

Tapken, Rahmel-Stein, and colleagues went on to demonstrate that clinical rescue, defined as survival and motor improvement, may coexist with incomplete molecular restoration within individual cell populations. Experimental studies in mice have demonstrated abnormalities of lymphoid organ architecture, hematopoiesis, and B cell development in SMA, with early SMN restoration preventing substantial components of this pathology (6). Conversely, Tapken, Rahmel-Stein, and co-authors found persistent immune abnormalities in adults with SMA who had been treated with nusinersen or risdiplam (4). Similarly, Lu et al. found NK cell and CD8^+^ T cell expansions in cerebrospinal fluid from individuals with untreated SMA, with unaltered protein/transcriptional profiles after nusinersen treatment (7). These persistent abnormalities could reflect developmentally fixed pathology, quantitatively insufficient SMN restoration, secondary effects of longstanding disease, or clinically inconsequential biological abnormalities (Figure 1B).

The critical question is whether residual biology has functional consequences for patients. The emerging generation of children treated in the first months of life offers a unique opportunity to distinguish among these possibilities (1). If an extraneuronal phenotype is secondary to severe neuromuscular disease, preventing motor disease should prevent the phenotype’s development. If it reflects a critical developmental requirement for SMN, treatment before that window may likewise prevent it. Conversely, if cells require sustained SMN expression throughout life, abnormalities may emerge despite excellent early motor outcomes. These early treatment cohorts may therefore reveal which components of SMA are developmental, secondary, reversible, or dependent on lifelong SMN insufficiency. And, most important, they may reveal which components have functional consequences as patients grow into adulthood.

Tapken, Rahmel-Stein, and colleagues have not yet demonstrated clinically meaningful immune dysfunction in SMA. Altered immune cell proportions and reduced SMN do not establish susceptibility to infection, impaired vaccine responses, autoimmunity, or pathological inflammation (4). This distinction should define the next phase of investigation. The goal should not simply be increasingly granular immune phenotyping, but linking immune phenotype to function and patient outcome. Longitudinal studies beginning before treatment could integrate cell-specific SMN and immune composition with vaccine responses, infection burden, lymphocyte function, inflammatory biomarkers, treatment exposure, and clinical outcomes.

## Do SMN-dependent immune alterations shape the response to therapy?

An additional consideration distinguishes the immune system from many other peripheral tissues in the context of SMA: Immunity may itself be affected by SMN deficiency while simultaneously shaping responses to adeno-associated virus (AAV) gene therapy. Jordan et al. showed that infants with SMA had immune exhaustion in CD8^+^ T cells, with suppression of inflammatory pathway activity prior to gene therapy and marked elevation after (8). It is unclear whether the elevated immune response is specific to the AAV9 vector itself, or if it is triggered by SMN expression or endogenous disease progression (6). Whether an SMN-deficient immune state influences AAV transduction, efficacy, toxicity, or durability remains unknown.

These questions become increasingly relevant as SMA therapies restore SMN through different mechanisms, routes, and anatomical distributions. Intravenous and intrathecal gene replacement, systemic SMN2 modification with risdiplam, and intrathecal SMN2 modification with nusinersen may confer similar motor benefit while producing different patterns of SMN restoration across cell types (1). This creates a natural experiment: Could therapies with comparable motor efficacy nevertheless generate different long-term systemic phenotypes because they restore SMN differently across tissues? Tapken, Rahmel-Stein, and co-authors showed that several immune cell populations in risdiplam-treated individuals were more similar to those of healthy controls than the same immune cell populations in nusinersen-treated individuals, an intriguing observation, given the systemic distribution of risdiplam (4). However, the small cross-sectional cohorts, nonrandomized treatment allocation, and differences in disease and treatment duration among participants preclude conclusions about differential immune rescue.

## Questions for the next era of SMA therapy

Tapken, Rahmel-Stein, and colleagues contribute more than another example of extraneuronal SMA. Their findings connect one of the oldest biological questions in SMA (what causes selective vulnerability to deficiency of a ubiquitous protein?) with one of its newest therapeutic challenges: What constitutes adequate correction after treatment? This is a question that can be posed broadly to the development and testing of all gene therapies, and SMA is unusually well suited to address this question. Its genetic cause is defined, SMN can be quantified, therapies restore the same protein through different mechanisms and routes, newborn screening enables presymptomatic intervention, and treated patients are now surviving long enough for residual biology to become apparent.

The framework for determining sufficient SMN restoration may ultimately require answers to five questions: Where is SMN restored? When is SMN restored? How much SMN does an individual cell require? What happens what that requirement is not met? Does the residual abnormality have clinical consequences?

The first therapeutic era of SMA established that increasing SMN could transform survival and motor function. The next must define adequate SMN restoration across each cell and across the lifespan.

## Manuscript information

### AI disclosure.

The authors declare that no AI tools were used in the original research for or writing of this article.

### Conflict of interest.

The author has declared that no conflict of interest exists.

## Figures and Tables

**Figure 1:**
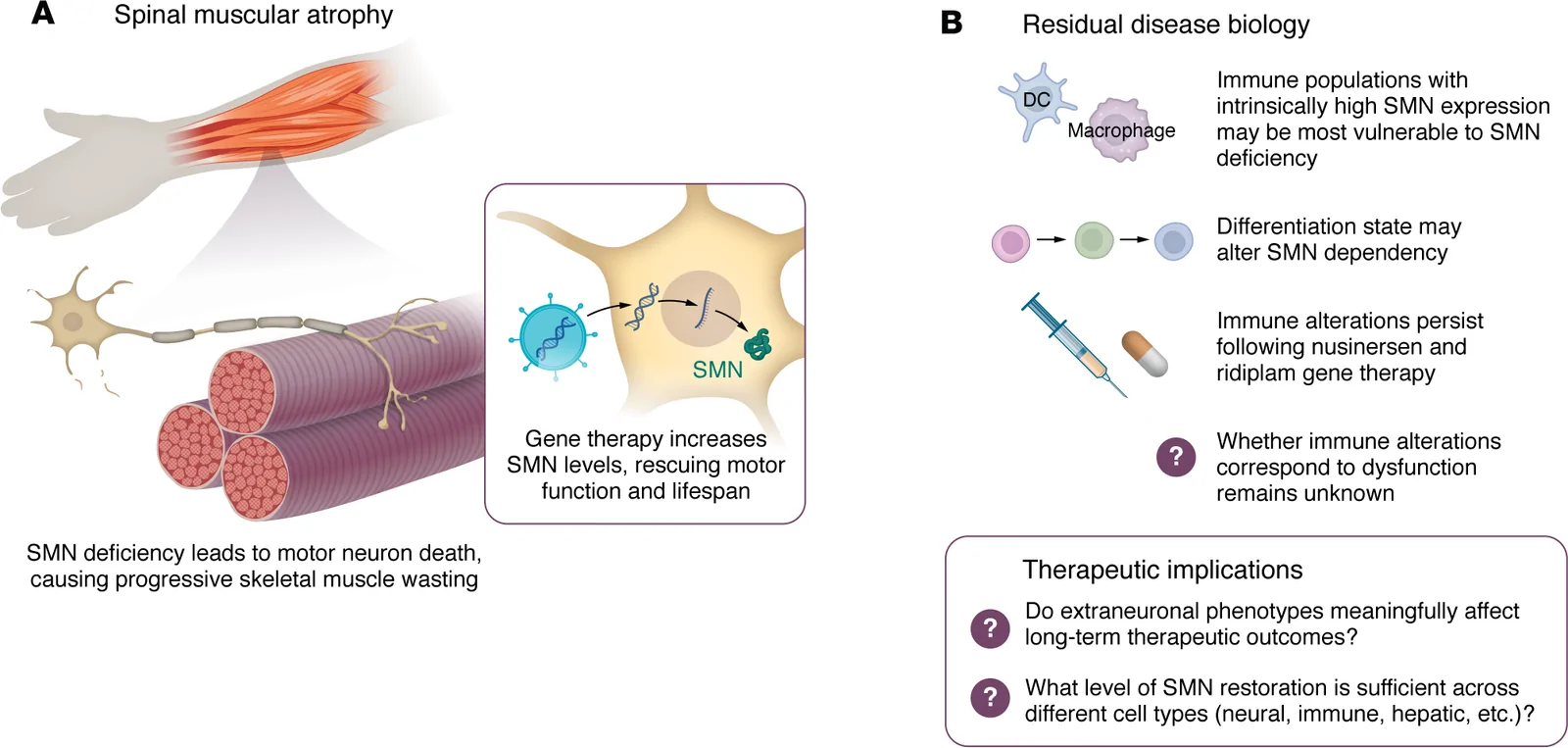
Immune alterations driven by SMN deficiency highlight complexities in long-term outcomes for treatment-modified SMA. (**A**) SMA is a neuromuscular disease caused by pathogenic mutations in the *SMN1* gene that lead to SMN deficiency and motor neuron death. The success of gene therapies including nusinersen, risdiplam, and onasemnogene abeparvovec to correct SMN deficiency in motor neurons has profoundly increased survival of patients with SMA. (**B**) Longer patient lifespans have revealed effects of SMN deficiency that extend beyond the neuromuscular system. Tapken, Rahmel-Stein, and co-authors (4) revealed that SMA was associated with alterations in immune cell populations that persisted after the completion of gene therapy. Their findings suggest that DCs and monocytes may have higher intrinsic requirements for SMN than other immune cells, rendering them more vulnerable to alteration in SMA. The authors’ findings add to the growing interest in extraneuronal phenotypes that may play important roles in long-term outcomes of gene therapy–modified SMA.

## Version 1 11/02/2026

Electronic publication
